# Smartphone Applications for Encouraging Asthma Self-Management in Adolescents: A Systematic Review

**DOI:** 10.3390/ijerph15112403

**Published:** 2018-10-29

**Authors:** Alaa Alquran, Katrina A. Lambert, Ambereen Farouque, Anne Holland, Janet Davies, Edwin R. Lampugnani, Bircan Erbas

**Affiliations:** 1 School of Public Health, College of Science Health and Engineering, La Trobe University, Bundoora 3083, Victoria, Australia; alaalmahmoud7@gmail.com (A.A.); k.lambert@latrobe.edu.au (K.A.L.); amber.farouque@gmail.com (A.F.); 2 Alfred Health Clinical School, Department of Rehabilitation, Nutrition and Sport, School of Allied Health, College of Science Health and Engineering, La Trobe University, Bundoora 3083, Victoria, Australia; A.Holland@latrobe.edu.au; 3 Faculty of Health, School‐Biomedical Sciences, Queensland University of Technology, Brisbane 4000, Queensland, Australia; j36.davies@qut.edu.au; 4 School of Biosciences, the University of Melbourne, Melbourne 3010, Australia; edwin.lampugnani@unimelb.edu.au

**Keywords:** asthma, smartphone, self-management, asthma control, adherence, self-efficacy

## Abstract

Adolescent asthma is still a major problem with poor adherence to treatment. Globally, adolescents are devoted users of smartphone technologies and app use in asthma self-management may improve adherence. The objective of this systematic review is to assess the feasibility and efficacy of mobile technology in improving asthma outcomes in adolescents. We conducted an extensive review of the peer-review literature of studies with populations consisting of children and adolescents under 18 years in seven bibliographic databases and Google Scholar. All study designs were considered. Quality assessment of included studies were independently assessed and reported. The search identified 291 articles; of the 16 eligible full-text papers, 8 met the review criteria, reporting two interventional, two qualitative and four observational studies. Samples ranged from 12 to 21 participants. Heterogeneity related to study design and the methods of the included studies prevented meta-analysis. Nevertheless, the intervention studies reported a positive effect of smartphone apps on asthma control, medication adherence and self-efficacy. Smartphone apps may be an effective asthma control tool especially among adolescents who are major users of smartphones; however, conclusions are limited by a lack of controlled trials and adequate sample sizes.

## 1. Introduction

For over a decade, mobile phones have been used as a tool for improving management of chronic illnesses including widespread health interventions of smoking cessation for respiratory diseases. Short message services (SMS) have been implemented for providing appointment reminders [1], improving medication adherence [2], and supporting asthma management [3]. Similarly, web-based eHealth interventions show improvement in chronic disease control [4] and patient empowerment [5].

Smartphone applications (apps) have the ability to combine the functionality of SMS and web-based eHealth solutions. The availability of inexpensive handsets and inclusion of mobile data in most service plans has led to an ever-increasing number of individuals using smartphones. In 2015, 73% of US adolescents used a smartphone [6], in Australia 80% of adolescents used a smartphone [7]. The ready availability of these devices has provided additional capacity and novel mechanisms for health-care delivery and self-management of major chronic diseases among adolescents.

Asthma is one of the most prevalent chronic diseases among adolescents in industrialized societies [8]. A steady increase in asthma prevalence globally has been reported by the World Allergy Organization [9], especially in countries such as Australia, UK, and the USA. The global burden of asthma is estimated to affect over 300 million people [8]. There are serious challenges in the day-to-day management of asthma, including medication adherence and symptom control.

As with any long-term chronic disease, asthma self-management forms an integral part of health care. While terminology is fluid [10], self-management can be defined as the tasks that individuals must undertake to live with their chronic condition, such as medical management, role management and emotional management. Self-management interventions encourage patients to actively participate in their care and increases their responsibility for controlling the symptoms and complications of their disease [11]. Strategies focus on education, empowerment, self-judgement and adherence to preventative strategies. The most common, though underutilised, self-management strategy in asthmatic children and adolescence is an “Asthma Action Plan” [12]. Self-efficacy, or the confidence in one’s abilities to self-manage, is another important component in the management of long-term chronic disease [10].

A recent review of publicly available asthma smartphone apps [13] looked at asthma self-management apps downloadable from the Google Play store and the Apple App store. They identified 38 “well-adopted” apps focusing on asthma self-management and 82 apps with less than 1000 downloads (Google Play store) or less than five reviews (Apple App store). Of the 38 well-adopted apps, 36 (95%) were free to download. Most of the apps were developed by private companies and independent developers, with university hospitals and health-related foundations developing four apps (11%, 4/38) and a governmental nonprofit body was responsible for one app (3%, 1/38). Despite this, almost half of the apps claimed that they involved health-care entities in the development process.

We conducted a systematic review to assess the feasibility and effectiveness of mobile phone apps in improving asthma self-management outcome measures among children and adolescents (e.g., asthma control tests, self-efficacy, and asthma medication adherence); we also sought to understand participants’ opinions about the role of mobile apps and monitoring technology in improving asthma management.

## 2. Materials and Methods

### 2.1. Eligibility Criteria and Definitions

This review was conducted according to the Preferred Reporting Items for Systematic Reviews and Meta-Analysis (PRISMA) guidelines (Supplement). We included human studies, published in English, with study populations consisting of children and adolescents aged less than 18 years. The participants in each study of this systematic review had a diagnosis of asthma. Studies must have focus on the use of a smartphone app for self-management of asthma or measurement of medication adherence and/or asthma symptoms. Interventions that used mobile phone technology solely to receive or deliver text messages were excluded. Studies that solely used mobile-phone-based internet were also excluded from this review. Applications that required additional technology such as sensors fitted to peak flow meters were excluded from this review.

### 2.2. Search Strategy

We used seven bibliographic databases: Medline (PubMed), Web of Science, Scopus, EMBASE, ProQuest Health, CINAHL (EBSCO), and Google Scholar (first 10 pages) to conduct the search. The search terms are listed in Table 1. Searches were also conducted into the reference lists from articles included in the literature review. Articles were limited to publication between 1 January 2007 and 30 June 2018.

### 2.3. Study Selection

After duplicate removal, the title and abstract of all papers returned by the search were assessed by two reviewers. Studies clearly not meeting the above eligibility criteria were excluded. The full text of the remaining papers was retrieved and critically assessed for inclusion in the review.

### 2.4. Data Extraction

The following data were extracted from each study: Study methods including type of study, study design, and details of control and comparison groups in intervention studies. The number of participants, age groups, and the country where the study was conducted; the definition of the app/downloaded software system or device used with a mobile phone; outcome measures and how the outcome data were obtained; estimated effect size with corresponding 95% confidence interval if quantitative statistical analysis was conducted. We have organised the results from intervention studies according to the effect of mobile phone use on the common outcomes including asthma control tests, self-efficacy, asthma medication adherence, and participant satisfaction with the interventions.

### 2.5. Quality Assessment

To conduct the quality assessment, we used an assessment tool that allowed assessment of studies that used both quantitative and qualitative methodology. We adapted a scale from many previously published sources that covered the areas relevant to the qualitative and quantitative studies [14]. A 12-question scoring tool (Yes, No) was used to assess the reporting quality, external validity, and bias. Two authors independently assessed each article (A.Q. and A.F.), and a consensus was reached after the researchers discussed their differences and sought input from a third author (B.E.) as required and came to an agreement.

## 3. Results

After a literature search of seven online databases, 561 records were identified. Removing duplicate records left 291 articles which were screened to further assess their applicability to the review. After the initial screening of the abstracts, 275 articles were excluded, and full manuscripts for 16 studies were assessed for inclusion criteria.

**Figure 1:**
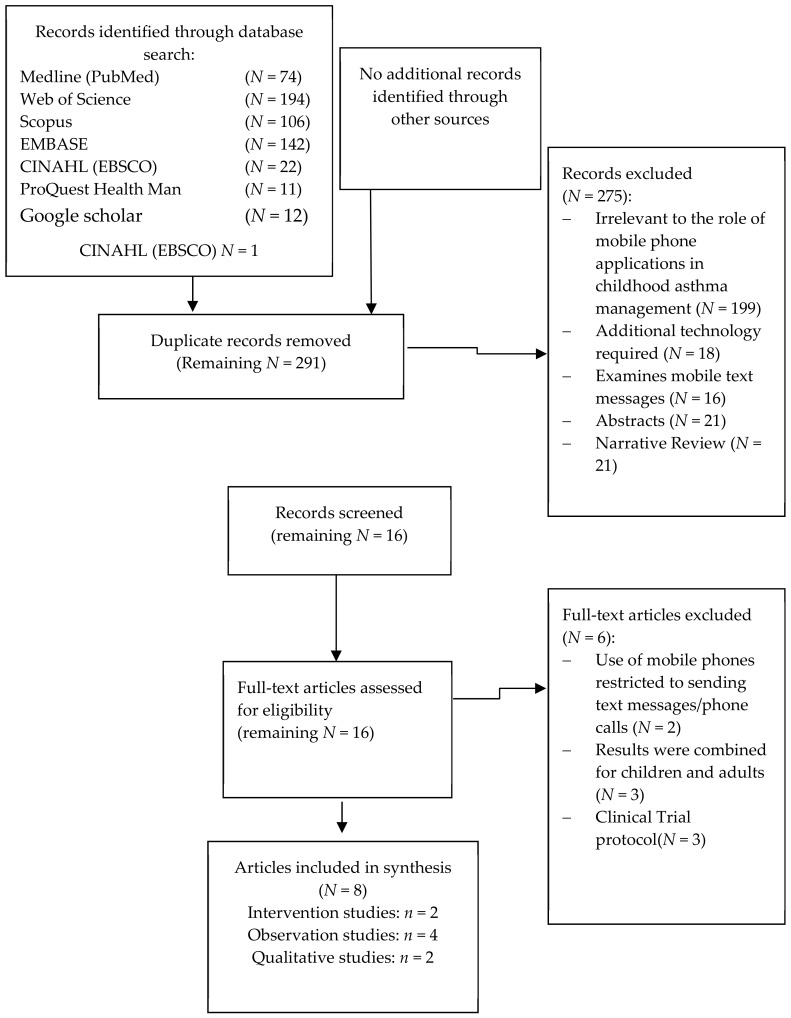
Preferred Reporting Items for Systematic Reviews and Meta-Analysis (PRISMA) flow chart of search methodology.

A further eight studies were excluded, use of mobile phones was restricted to sending text messages/phone calls in two papers [15,16], results were combined for children and adults in three [17,18,19], and three were trial protocols [20,21,22]. After applying the exclusion criteria, eight studies remained on the use of phone technology in asthma self-management among asthmatic children and adolescents (Figure 1).

Almost all studies were of reasonable quality as the methods of recruitment were clear, characteristics of the participants clearly defined, method of data collection were clearly defined, and data collected systematically. However, most suffered from small sample size given that most were pilot studies (Table S1).

### 3.1. Results from Intervention Studies

We identified two intervention studies both conducted in the US with similar age groups [23,24] (Table 2). Both studies focused on socially disadvantaged groups, participants in one study were an urban minority group of African Americans [24], whilst the other was conducted in a mixed population (60% African American) where 90% of participants had state-issued medical insurance [23]. Both studies were small pilot trials (*n* = 12 and *n* = 20, respectively) of applications designed by the researchers. Asthma self-management was measured by multiple outcomes in both studies (Table 3).

#### 3.1.1. Asthma Control

Improvements in asthma control test (ACT) scores were shown in both intervention studies. A study of outpatients aged 12–17 years with persistent asthma from Arkansas Children’s Hospital [23] documented an increase in median ACT scores increased from 20 during pre-intervention to 21.5 during post-intervention for all participants (*n* = 18). However, amongst the nine participants with uncontrolled asthma the median ACT scores increased from 16 (13–17) at baseline to 18 (16–23) post-intervention (*p* = 0.03). Consistent results were documented in an intervention study of 12 participants with persistent asthma between the ages of 11 and 16 [24]. Although two of the participants did not complete the final assessment at the end of the study (week eight), their final observations were used for the analysis. The participants’ median ACT scores improved from 18 at baseline to 23 in the 8th week of the study.

#### 3.1.2. Self-Efficacy

In Burbank et al.’s study [23], the child asthma self-efficacy score was calculated using a questionnaire to measure the children’s self-efficacy regarding asthma attack prevention and attack management. While the median asthma attack prevention self-efficacy score significantly increased from 34 at baseline to 36 after the intervention (*p* = 0.04), attack management self-efficacy score did not improve (*p* = 0.44). The median total self-efficacy score did not increase over the course of the intervention (pre: 60.5, post: 62, *p* = 0.13).

#### 3.1.3. Asthma Medication Adherence

Mosnaim and colleagues [24] measured percent daily inhaled corticosteroid (ICS) adherence using an electronic medication monitor placed on the participants’ medication. They reported that the mean level of use increased from 19% at baseline to 50% (intent-to-treat) and 67% (observed) after eight weeks of using the app. Further, the percentage of participants who met the target ICS adherence (>50%) improved from 8% at baseline to 58% at week eight.

#### 3.1.4. Participant Satisfaction

Burbank and colleagues [23] found high participant satisfaction with the Asthma Action Plan (AAP) application, and 93% of participants reported that they would adhere to their medication better while using the AAP application.

### 3.2. Results from Observational Studies

Three observational studies assessed asthma self-management apps (Table 4), one which has been reported on twice [25,26]. Again, all studies were conducted in the USA amongst adolescents.

An observational study in North Carolina which involved 20 adolescents with persistent asthma aged 12–16 years old as well as their caregivers has been discussed twice [25,26]. The adolescents used two asthma apps for more than one week. The primary outcome measures in this study included the following: the participants’ asthma goals; the adolescents’ technology use; the number of participants who reported the usefulness of each feature in the apps; and the effect of app features on self-observation, self-judgment, and self-reaction.

According to the participants, the most useful app features included the appointment reminders (44%), the medication reminders (56%), the peak flow monitor (50%), the diary feature (69%), the trigger and symptoms feature (75%), the charging feature (69%), and the allergies and emergency plan (63%). For the 16 participants in the telephone interview, 15 felt that they became more involved in asthma self-management after using the apps [25].

Interviews after using the apps for the week [26] discussed using communication with others using asthma applications. Many adolescents discussed how knowledge of asthma control could be shared by syncing the app with their caregiver. Ten (63%) adolescents said they wanted their friends involved in an asthma app and the majority (88%) would use an app to send information to their medical provider.

Teens in Schneider and colleagues’ study [27] also used existing apps for one week before providing feedback. The two apps investigated were asthma self-management apps available for download by the general public which were tailored to teens. Features identified as useful were the ability to directly communicate with the medical provider, to personalise features and information, medication logs, visual aids in the form of graphs and charts, and an asthma journal. Additional features that were suggested included a prompt to use the app, reminders to take medication or purchase refill, and a fun factor in the form of asthma-related games and customisable design.

Odom and Christenberry [28] describe the development of a digital asthma action plan application. A short survey of 12 children with asthma, aged 12 to 19, reported that the developed application was easy to use and could be used without any written instruction. Furthermore, they believed that the use of the app simplified their asthma action plan. The authors did not give an indication of how long participants were using the app before giving their feedback.

### 3.3. Results from Qualitative Studies

Two qualitative studies were conducted on focus groups in the Netherlands [29] and the USA [30] (Table 5).

Koster et al. [29] used focus groups of 21 asthmatic adolescents. In this study, 10 adolescents reported that forgetting was the reason for non-compliance, and 12 stated that reminding them to take their medication was their parents’ responsibility. With regard to potentially enhancing compliance, 11 adolescents reported that they kept the medication in a fixed, predetermined place. Lastly, five (four older and one young) adolescents reported that mobile apps were a potential means by which to improve compliance.

Similarly, the focus groups conducted by Panzera and colleagues [30] reported forgetting to take their daily medication or to take inhalers with them when they leave home as a major barrier to asthma control. The focus groups consisted of 18 adolescents with asthma (aged 13–19 years) and their caregivers. The groups indicated that a digital media product capable of tracking conditions, triggers, and related asthma activities could help improve asthma control. A solution which included reminders for medication usage was viewed positively.

## 4. Discussion

We conducted this systematic review to assess the role of using mobile phone technology—namely, asthma management apps or interactive software systems—in improving conditions associated with asthma management among asthmatic children and adolescents. A combination of both quantitative and qualitative studies were synthesized. Overall, the results of the quantitative studies show limited evidence that the use of a smartphone app may have a positive effect on asthma symptom scores, self-efficacy asthma scores, and asthma medication adherence. Furthermore, the qualitative studies concluded that the mobile phone application could improve and facilitate asthma self-management among children and adolescents.

Smartphones could play an important role in improving some aspects of patient health outcomes. Medical smartphone apps were owned by 16.9% of teenagers in New York in 2014 (*n* = 148) [31]. The number of asthma apps available for download via Google Play or Apple Apps has varied from 94 in 2011 to 147 in 2013 [32] to 120 in 2016 [13]. This wide variety and dynamic aspect of apps affects their suitability for use in clinical settings of asthma self-management as they are not usually supported by clinical testing [13]. It is important to stay updated regarding the number of qualified asthma apps available and to restrict use of the ineffective asthma apps, which negatively affect accreditation of effective apps for use in asthma self-management internationally [32]. This could illustrate why, although there are many asthma apps, we found only two intervention studies that used asthma self-management apps as an intervention among adolescents with asthma.

There is a great need for improving asthma patients’ adherence to medication [33], as low adherence to taking prescribed medications is a major hurdle in treating many chronic diseases, especially among adolescents who demonstrate “at risk” behaviours. Our review indicates a potential improvement to be gained in patients’ medication adherence because of smartphone apps for asthma self-management. This was a key theme highlighted in the qualitative studies, and improvement was noted in one of the intervention studies [24]. The results also indicate that there is a potential improvement in asthma control to be gained from asthma self-management apps. Adolescents’ self-efficacy in asthma management was only investigated by one study [23], more studies will be required to conclude if mobile technology can improve self-efficacy.

Our ability to draw inference from these results is limited. The studies synthesised in this review were pilot studies with small sample sizes ranging from *n* = 12 [24] to *n* = 21 [29]. The intervention studies were short-term, just eight weeks long, and the observation studies were conducted after the participants were using the apps for one week. There is a need for assessment of the smartphone app usage among adolescents for an extended period of time, to see if the improvements in self-management are sustained. Longer studies are also needed to assess the feasibility of long-term use of the app. Furthermore, none of the studies were randomised controlled trials, which decreases confidence in the observed effects.

Previous systematic reviews have been conducted to analyse digital interventions for improving self-management of asthma, including website-based interventions, mobile phone technology, text messaging, and smartphone apps with mixed results [34,35,36]. Nickels and Dimov [36] reviewed the evidence for the effectiveness of technological interventions among adolescent asthmatics. They concluded that there was no consistent evidence of the effectiveness of such technologies. At the time of conducting their review in 2012, they did not find any studies that explored the use of smartphone apps. A Cochrane review in 2013 [34] looked at self-management interventions for patients with clinician-diagnosed asthma delivered via smartphone apps. They did not find any studies related to adolescents, locating only two randomised control trials among adults that meet their inclusion criteria. The authors concluded that this was insufficient to advise on the use of smartphone and tablet computer apps for the delivery of asthma self-management programmes. However, in a systematic review conducted by Morrison and colleagues [35] in 2014, the authors concluded that digital self-management interventions could have the promised beneficial effect on some outcomes (e.g., self-care, quality of life, and medication use) among children and adults with asthma. Again, no asthma self-management interventions delivered by smartphone app amongst adolescents were identified.

There have been an increasing number of studies developing and testing the feasibility and efficacy of smart applications in asthma care in adolescents since the Morrison and colleagues review in 2014. Therefore, this review paper is a timely and important attempt to evaluate and reflect on the current state of science in the field of mobile technology and its application for paediatric asthma self-management. Our paper is strengthened by the inclusion of both quantitative and qualitative studies in the review.

Few studies, considered as pilots and with limited sample size, have focused on the use of smartphone applications on treatment adherence and asthma management among children and adolescents. Major gaps exist in understanding whether these technological interventions are relevant and meet the needs of high-risk asthmatics. Adolescents are the major, if not the leading, group compared to the general population that grasps, understands, and uses this new technology as part of their everyday lives. Asthma is a condition that is highly prevalent in this group and is poorly managed. By using the one to address the other, new technological tools are needed that may change “at risk” behaviours and thus improve quality of life. Many studies are needed to demonstrate efficacy of this approach in managing these chronic conditions. Moreover, many triggers are seasonal. Studies are needed to assess uptake of these smart technologies during peak seasonal periods such as high pollen days and address treatment adherence and day-to-day management.

## 5. Conclusions

The main finding of this review is the dearth of evidence-based apps, as opposed to the large number of smartphone apps on the market for the general population of asthmatics designed to monitor self-management and medication use. Although our aim was to assess the effectiveness and feasibility of smartphone apps, we cannot reach a specific conclusion on the effect because of the small number of included articles on using smartphone apps in improving asthma management and the heterogeneity between the included articles. Based on our findings, smartphone apps could be considered an effective health intervention. However, future studies should investigate the role of such technology in randomised control trials among larger sample sizes.

## Figures and Tables

**Table 1:** Ovid search strategy.

Search No.	Search Terms
1	Child * OR pediatric OR paediatric OR adolescents OR teen * OR school OR PEDIATRICS/
2	smart OR mobile OR phone OR CELL PHONE/
3	application OR app *
4	asthma OR ASTHMA/
5	self-manage * OR control OR self-efficacy OR adherence OR medication OR compliance OR observation OR judgement OR “action plan”
6	1 AND 2 AND 3 AND 4 AND 5

**Table 2:** Characteristics of the intervention studies.

Author	Study Population; Location	Age Group (Sample Size)	Socially Disadvantaged Groups	Description of the App	Measures and Outcomes
Burbank et al. [22]	Outpatient paediatric specialty clinic; Arkansas, USA	12−17 years (*N* = 20)	- 60% were minorities - 90% had state-issued medical insurance	Asthma Action Plan (AAP). This sends two daily notifications to the participant. The first one prompts for the recording of asthma symptoms and peak flow, and the second one is a medication reminder. It also provides guidance and advice every week via push notification.	- Usage rate of application; - Participant satisfaction; - Asthma control test scores (pre-and post-intervention); - Self-efficacy scores (pre- and post-intervention)
Mosnaim et al. [23]	Rush Paediatric Primary Care Centre, Chicago, Illinois, USA	11−16 years (*N* = 12)	African-American population (urban minority group).	Mobile Adolescents’ Disease Empowerment and Persistency Technology (M-ADEPT): - Reminders for participants to take their ICS medication - Basketball game (immediate encouragement) for taking inhaled corticosteroid (ICS) - Positive text messages for each puff of ICS taken	‘Efficacy’ defined as: - asthma control test (ACT) score should be improved by ≥3 points after intervention; - ICS use should be ≥50%. Main measures: - ACT scores (pre- and post- intervention); - ICS adherence.

**Table 3:** Outcomes measured by the included intervention studies.

Author	Asthma Control Tests	Self-Efficacy	Asthma Medication Adherence	Participant Satisfaction
Burbank et al. [22]	Median ACT scores increased from 20 to 21.5. ACT scores for uncontrolled asthma (*N* = 9) increased from 16 to 18 (*p* = 0.03).	Median total self-efficacy increase from 60.5 to 62 for all participants (*p* = 0.13)		93% of participants reported their satisfaction.
Mosnaim et al. [23]	58% of the participants achieved three points in their ACT scores by week eight of the study. Median ACT increased from 18 to 23.		Median ICS adherence increased from 19% to 67 %.Short-acting beta 2-agonist decreased from median of three to zero.	

**Table 4:** Characteristics of the observational studies.

Author	Study Population and Location	Age Group (Sample Size)	Other Variables (e.g., Sex, Socially Disadvantaged Groups)	Measures and Outcomes
Carpenter et al. [25], Roberts et al. [26]	Adolescents and their caregivers from two paediatric practices located in an urban area of North Carolina, USA	Age 12−16 (*N* = 20)	- Males = 11 - Females = 9 - White = 9 - Black = 8 - Annual household income: <$25,000	1. Participants’ asthma management goals 2. Adolescent technology use 3. Some participants reported the usefulness of each feature in the apps 4. The effect of app features on self-observation, self-judgment and self-reaction
Odom and Christenberry [28]	Patients returning for follow-up care at the allergy/immunology practice with a previous diagnosis of asthma, Tennessee, USA	Age 12–19 (*N* = 12)	- Female: 60%	1. Ease of use 2. Use without written instructions 3. Expectation of the app features
Schneider et al. [27]	Adolescents with daily asthma maintenance medications, Tampa Bay, FL, USA	Age 13–18 (*N* = 16)	- Not specified	1. Usefulness of features 2. Additional feature suggestions 3. Interface and app operation feedback

**Table 5:** Characteristics of the qualitative studies.

Author	Study Population andLocation	Age Group and Sample Size	Other Variables (e.g., Sex, Socially Disadvantaged Groups)	Measures and Outcomes
Koster et al. [29]	Two study populations: 1. An online group from the Utrecht Pharmacy Practice Network for Education and Research, the Netherlands in December 2013 2. A face-to-face group from the Department of Paediatrics at the Medical Centre Leeuwarden, the Netherlands in March 2014.	12−16 years: (*N* = 21) - Online group (*N* = 14) - Face-to-face group (*N* = 7)	Online group: - Males = 8 - Females = 6 Face-to-face group: - Males = 2 - Females = 5	1. Adherence: This study clarifies the reasons for not taking medication at the proper times 2. Role of parent(s) 3. The solution that supports medication use 4. The effect of mobile phone applications on medication use
Panzera et al. [30]	Teens with asthma and their parent-caregivers who attended pediatric pulmonary clinics, Florida USA	13–19 years of ages *N* = 18		1. Adherence: This study clarifies the reasons for not taking medication at the proper times 2. Potential role of digital media 3. Potential role of social media

## Supplementary Materials

The following are available online at http://www.mdpi.com/1660-4601/15/11/2403/s1.
