# Supplementary material for: Smartphone Applications for Encouraging Asthma Self-Management in Adolescents: A Systematic Review
Source: Int J Environ Res Public Health. 2018 Oct 29;15(11):2403. doi: 10.3390/ijerph15112403 (PMC6266660; doi:10.3390/ijerph15112403)
Supplement: Supplementary file 1 [file ijerph-15-02403-s001.pdf]

Table S1. Quality assessment.

**Study Assessment Question**	***Burbank et al.* [22]**	***Carpenter et al.* [25]**	***Koster et al.* [29]**	***Odom et al.* [28]**	***Panzera et al.* [30]**	***Roberts et al.* [26]**	***Schneider et al.* [27]**
1. Is the research question clearly stated?	Yes	Yes	Yes	Yes	Yes	Yes	Yes
2. Are the criteria for selecting the sample clearly defined?	Yes	Yes	Yes	Yes	Yes	Yes	Yes
3. Is the method of recruitment clear?	Yes	Yes	Yes	Yes	Yes	Yes	Yes
4. Are the characteristics of the sample adequately defined?	Yes	Yes	Yes	Yes	Yes	Yes	Yes
5. Is the final sample adequate and appropriate?	Yes	Yes	No	No	Yes	Yes	Yes
6. Was the method for collecting data adequately described?	Yes	Yes	Yes	Yes	Yes	Yes	Yes
7. Was the data collected systematically?	Yes,	Yes	Yes	Yes	Yes	Yes	Yes
8. Was the relationship between the researcher and the participant explicit?	Yes	Yes	Yes	Yes	Yes	Yes	Yes
9. Were the methods used in the data analysis appropriate and designed to minimize bias?	Yes	Yes	Yes	Yes	Yes	Yes	Yes
10. Is evidence provided in support of the analysis?	Yes.	Yes	Yes	Yes	Yes	Yes	Yes
11. Is there evidence of efforts to establish validity?	No	No	No	No	No	Yes	No
12. Were the conclusions drawn appropriate given the results?	Yes	Yes	Yes	Yes	Yes	Yes	Yes
